# Advanced control parameter optimization in DC motors and liquid level systems

**DOI:** 10.1038/s41598-025-85273-y

**Published:** 2025-01-09

**Authors:** Serdar Ekinci, Davut Izci, Mohammad H. Almomani, Kashif Saleem, Raed Abu Zitar, Aseel Smerat, Vaclav Snasel, Absalom E. Ezugwu, Laith Abualigah

**Affiliations:** 1 Department of Computer Engineering, Batman University, Batman, 72100 Turkey; 2 Applied Science Research Center, Applied Science Private University, Amman, 11931 Jordan; 3 Department of Mathematics, Facility of Science, The Hashemite University, P.O box 330127, Zarqa, 13133 Jordan; 4 Department of Computer Science & Engineering, College of Applied Studies & Community Service, King Saud University, Riyadh, 11362 Saudi Arabia; 5 Faculty of Engineering and Computing, Liwa College, Abu Dhabi, United Arab Emirates; 6 Faculty of Educational Sciences, Al-Ahliyya Amman University, Amman, 19328 Jordan; 7 Centre for Research Impact & Outcome, Chitkara University Institute of Engineering and Technology, Chitkara University, Rajpura, 140401 Punjab India; 8 Department of Biosciences, Saveetha School of Engineering, Saveetha Institute of Medical and Technical Sciences, Chennai, 602105 India; 9 Research Center, Mazaya University College, Nasiriyah, Iraq; 10 Faculty of Electrical Engineering and Computer Science, VŠB-Technical University of Ostrava, Poruba-Ostrava, 70800 Czech Republic; 11 Unit for Data Science and Computing, North-West University, 11 Hofman Street, Potchefstroom, 2520 South Africa; 12 Computer Science Department, Al al-Bayt University, Mafraq, 25113 Jordan

**Keywords:** Mountain Gazelle optimizer, PID controller, Parameter estimation, DC motor speed regulation, Liquid level control, Chemical engineering, Electrical and electronic engineering

## Abstract

In recent times, there has been notable progress in control systems across various industrial domains, necessitating effective management of dynamic systems for optimal functionality. A crucial research focus has emerged in optimizing control parameters to augment controller performance. Among the plethora of optimization algorithms, the mountain gazelle optimizer (MGO) stands out for its capacity to emulate the agile movements and behavioral strategies observed in mountain gazelles. This paper introduces a novel approach employing MGO to optimize control parameters in both a DC motor and three-tank liquid level systems. The fine-tuning of proportional-integral-derivative (PID) controller parameters using MGO achieves remarkable results, including a rise time of 0.0478 s, zero overshoot, and a settling time of 0.0841 s for the DC motor system. Similarly, the liquid level system demonstrates improved control with a rise time of 11.0424 s and a settling time of 60.6037 s. Comparative assessments with competitive algorithms, such as the grey wolf optimizer and particle swarm optimization, reveal MGO’s superior performance. Furthermore, a new performance indicator, ZLG, is introduced to comprehensively evaluate control quality. The MGO-based approach consistently achieves lower ZLG values, showcasing its adaptability and robustness in dynamic system control and parameter optimization. By providing a dependable and efficient optimization methodology, this research contributes to advancing control systems, promoting stability, and enhancing efficiency across diverse industrial applications.

## Introduction

In recent years, the field of control systems has witnessed tremendous developments. These advancements have been driven by the growing complexity and diversity of industrial applications, such as robotics, automation, and process control^1–3^. As a result of the fact that efficient control of dynamic systems is essential for reaching the desired level of performance and stability, the optimization of control parameters has been a key focus of research^4–9^.

Among the various approaches to control system optimization, model predictive control (MPC) has garnered attention for its ability to handle multi-variable systems and predict future behaviors. Model predictive control has been effectively applied in flexible hybrid microgrids with plug-and-play capabilities and energy management of hybrid microgrids using switched auto-regressive neural control^10,11^. Despite its advantages, model predictive control often faces challenges in real-time implementation due to its computational complexity.

To address these limitations, metaheuristic optimization algorithms have emerged as efficient alternatives^1^. These algorithms provide simpler implementations, faster convergence, and robust performance in diverse applications. In recent years, several metaheuristic techniques have been explored, including the grey wolf optimizer^12^, particle swarm optimization^13^, and the Henry gas solubility optimization^14^. Building on these advancements, this study introduces the mountain gazelle optimizer (MGO)^15^as it has shown promise due to its ability to emulate the agile movements and optimization strategies of mountain gazelles^16^.

In this paper, we propose a novel approach for the control of two distinct systems: a direct current (DC) motor^17^and a three-tank liquid level system^18^. Our focus lies in optimizing the control parameters of both systems using the MGO. We leverage the inherent characteristics of the MGO to fine-tune the parameters of a proportional-integral-derivative (PID) controller^19–22^ for each system, aiming to achieve superior performance.

DC motors are widely used in numerous industrial applications, making their control a crucial aspect of many systems^23–25^. To optimize the control parameters for the DC motor, we compare the performance of the proposed MGO-based approach with other competitive algorithms as a novel and efficient approach alternative to the previously reported ones^26–29^. Specifically, we consider the grey wolf optimizer^30^, atom search optimization^31^, improved model of marine predator algorithm^32^, and Henry gas solubility optimization^33^. By employing the integral of time multiplied by absolute error (ITAE) performance index^34^ as a cost function, we assess the time domain performance metrics, including overshoot, rise time, settling time, and peak time. The results demonstrate that the MGO-based approach achieves remarkable performance metrics, highlighting its efficacy in controlling DC motors.

Controlling the level of liquid in a variety of industrial processes, such as chemical plants and water treatment systems, is a vital component^35–37^. The purpose of this research is to expand the application of the MGO-based strategy to the control of a three-tank liquid level system. We propose this approach as an innovative and efficient alternative to the approaches that have been published in the literature^38–42^. To evaluate the effectiveness of our proposed method, we compare it against other competitive algorithms. Particle swarm optimization^43^, hybrid differential evolution and particle swarm optimization with an aging leader and challengers^43^, covariance matrix adaptation evolution strategy^44^, and arithmetic optimization algorithm with Harris hawks optimization^44^ serve as benchmarks for comparison. The ITAE performance index is again utilized as a cost function to assess the system’s time domain performance. The results demonstrate that the MGO-based approach outperforms the competing algorithms, indicating its efficacy in controlling three-tank liquid level systems.

In addition to the ITAE performance metric, we also introduce another performance indicator called $\:ZLG$^45^ as a time domain performance metrics-based approach. This indicator allows for a more comprehensive evaluation of control quality. We demonstrate that the proposed MGO-based approach consistently achieves lower $\:ZLG$ values when compared to the competitive algorithms. This underscores the versatility and robustness of the MGO in controlling dynamic systems and optimizing control parameters.

To briefly summarize, the purpose of this study is to demonstrate the innovative and better strategy that is shown in this paper for optimizing the control parameters of three-tank liquid level systems as well as DC motors by utilizing the MGO algorithm. The effectiveness of our proposed methodology in achieving superior control performance is demonstrated by the fact that it makes use of agility and optimization strategies that are inspired by mountain gazelles. We provide compelling evidence of the superiority of the MGO-based approach in terms of various performance indicators by conducting extensive comparisons with a variety of competitive algorithms. This allows us to make a significant contribution to the field of control systems by providing a dependable and effective optimization methodology. The MGO-based strategy that has been proposed opens up new avenues for the development of control systems. This is accomplished by improving the performance, stability, and efficiency of dynamic systems in a variety of industrial applications.

The remainder of this paper is structured as follows: Sect. 2 provides an overview of the MGO, including its behavioral inspiration and mathematical formulation. Section 3 presents the case studies, focusing on the application of the proposed method to a DC motor speed regulation system and a three-tank liquid level system. Section 4 details the implementation of MGO for PID controller parameter optimization, including the simulation setup and problem formulation. Section 5 discusses the simulation results and compares the MGO-PID controller’s performance with benchmark algorithms, emphasizing its efficacy and robustness. Finally, Sect. 6 concludes the paper by summarizing the key findings and highlighting potential avenues for future research.

## Mountain Gazelle optimizer

The MGO is an algorithm inspired by the behavior of mountain gazelles, which exhibit territorial, maternal, bachelor male herd, and migration behaviors^15^. These behaviors are expressed mathematically and employed to generate subsequent generations of gazelles. The most adept gazelles are retained within the overall population, whereas those that are feeble or aged are eliminated.

The mathematical model of the MGO basically incorporates four factors: solitary territorial males, maternity herds, bachelor male herds, and migration for food search^46,47^. Each gazelle in the algorithm can join one of three herds: solitary territorial males, maternity herds or bachelor male herds. New gazelles have the potential to be born into any of these herds. In the MGO algorithm, the optimum solution is represented by the adult male gazelle situated within the territory of a herd. Since male bachelor herds cannot procreate or control female gazelles, about one-third of the population is estimated to have the lowest cost among other modeling options. Upon reaching maturity, male mountain gazelles establish and defend solitary territories, engaging in fierce battles for dominance and access to females. This territorial behavior is modeled as:

1 $$\:TSM=malegazelle-\left|\left(ri1\times\:BH-ri2\times\:X\left(t\right)\right)\times\:F\right|\times\:Cofr$$

Here, $\:malegazelle$ represents the global best position, $\:ri1$ and $\:ri2$​ are random integers (1 or 2), and $\:BH$, $\:F$, and $\:Cofr$ are computed as follows:

2 $$\:BH=Xra\times\:\lfloor r1 \rfloor+Mpr\times\:\lceil r2 \rceil,\:ra=\left\{\left|\frac{N}{3}\right|\dots\:N\right\}$$

3 $$\:F=N1\left(D\right)\times\:exp\left(2-Iter\times\:\left(\frac{2}{MaxIter}\right)\right)$$

4 $$\:Cofr=\left\{\begin{array}{c}\left(a+1\right)+r3,\\\:a\times\:N2\left(D\right),\\\:r4\left(D\right),\\\:N3\left(D\right)\times\:N4\left({D}^{2}\right)\times\:\text{cos}\left(\left(r4\times\:2\right)\times\:N3\left(D\right)\right)\end{array}\right.$$

where $\:a=-1+Iter\times\:\left(-1/MaxIter\right)$, $\:Xra$​ is a random position within $\:ra$, $\:Mpr$​ is the average number of search agents, $\:N1$, $\:N2$, $\:N3$, and $\:N4$​ are random values from a normal distribution, and $\:r1$, $\:r2$ ,$\:r3$, $\:r4$​ are random numbers between 0 and 1. Female gazelles in maternity herds give birth to males, with young males competing for access to them. This behavior is represented by:

5 $$\:MH=\left(BH+Cof2,r\right)+\left(ri3\times\:malegazelle-ri4\times\:Xrand\right)\times\:Cof3,r$$

Here, $\:Cof2,r$ and $\:Cof3,r$ are computed as in Eq. (4), $\:ri3$ and $\:ri4$​ are random integers (1 or 2), and $\:Xrand$ is a random gazelle’s position. Male gazelles engage in intense battles for territory and females, modeled as:

6 $$\:BMH=\left(X\left(t\right)-D\right)+\left(ri5\times\:malegazelle-ri6\times\:BH\right)\times\:Cofr$$

where $\:D=\left(\left|X\left(t\right)\right|+\left|malegazelle\right|\right)\times\:\left(2\times\:r6-1\right)$, $\:X\left(t\right)$ is the position vector of the gazelle at the current iteration, $\:ri5$ and $\:ri6$​ are random integers (1 or 2), and $\:r6$​ is a random number between 0 and 1. Gazelles continuously search for food, exhibiting remarkable speed and agility. This behavior is captured as:

7 $$\:MSF=\left(ub-lb\right)\times\:r7+lb$$

Here, $\:ub$ and $\:lb$ denote the upper and lower bounds of the problem, and $\:r7$​ is a random integer between 0 and 1. The mechanisms $\:TSM$, $\:MH$, $\:BMH$, and $\:MSF$ generate new generations. Each generation undergoes sorting, preserving top-performing gazelles while removing weaker ones. The dominant male gazelle in the population is identified as the superior solution.

**Fig. 1:**
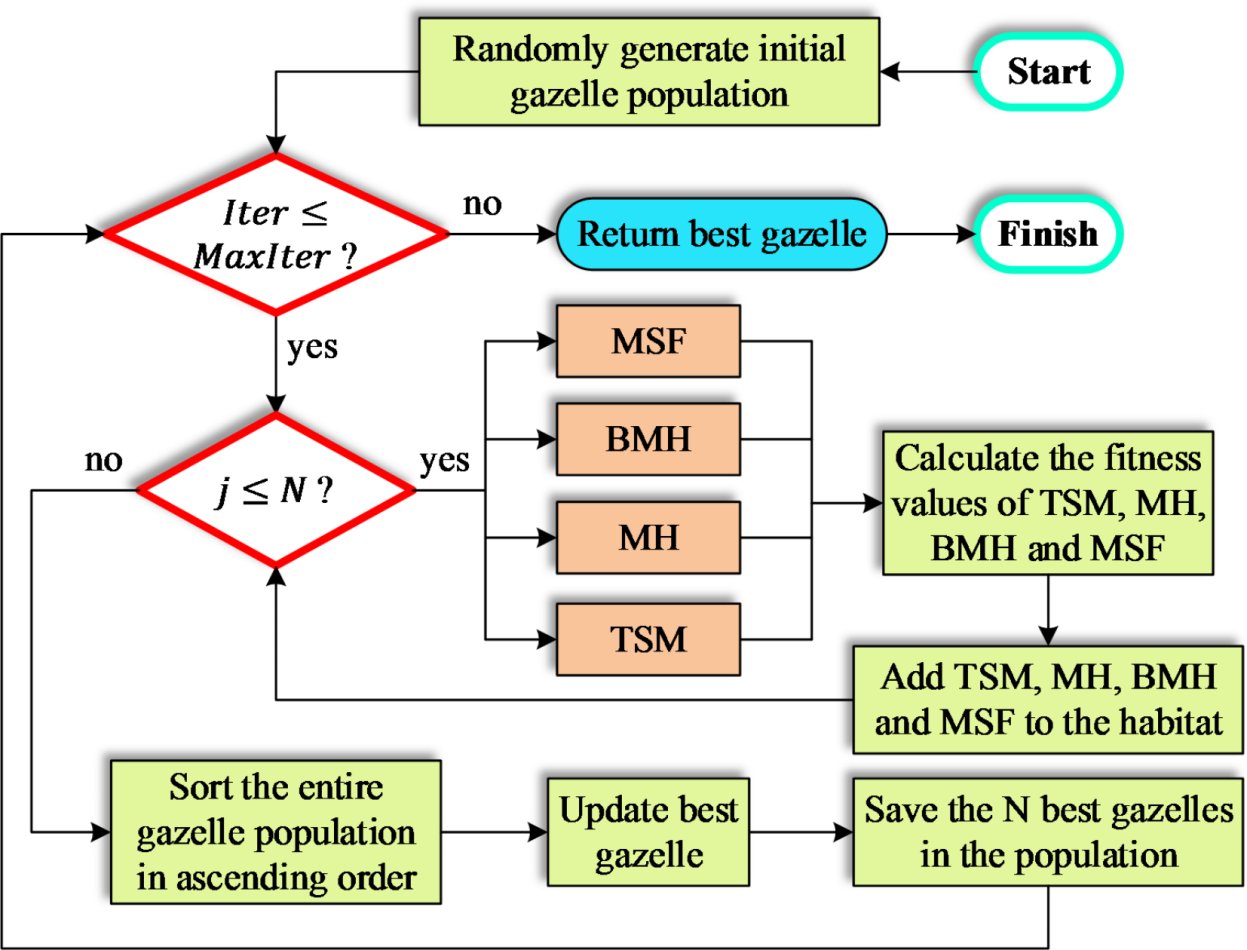
Flowchart of MGO algorithm.

The flowchart presented in Fig. 1 outlines the sequential steps of the MGO. As demonstrated, the MGO operates through four distinct behavioral mechanisms. The process begins with the initialization phase, where a population of gazelles is created, with each individual representing a potential solution. This population is divided into different herds, including solitary males, maternity herds, and bachelor male herds. The behavior modeling phase encompasses four key mechanisms: territorial behavior, where dominant males establish territories and compete for females to drive exploration; maternity behavior, where female gazelles contribute to population diversity by producing offspring; bachelor male competition, where young males compete to gain territories or access to females, enhancing exploitation; and food search migration, where gazelles explore new regions in the solution space in search of resources. Following these behaviors, the evaluation and selection phase assesses the fitness of each solution using a defined objective function, retaining the fittest individuals and eliminating weaker ones. Finally, the convergence check ensures that the population is iteratively updated until a predefined criterion, such as the maximum number of iterations or achieving a satisfactory fitness value, is met. By effectively balancing exploration and exploitation, the MGO demonstrates robust optimization performance.

## Case studies

In this section, we explore the application of the MGO in optimizing PID controller parameters for two distinct systems: a DC motor speed regulation system and a three-tank liquid level control system. The case studies are designed to assess the versatility and effectiveness of the proposed optimization approach. For the DC motor, the focus is on achieving precise and efficient speed regulation, which is critical for various industrial applications. Similarly, the three-tank system emphasizes maintaining desired liquid levels despite nonlinear interactions and dynamic disturbances. Through detailed modeling and comparative analysis with established algorithms, these case studies demonstrate the capability of MGO to enhance system performance under varying operational conditions.

### DC motor speed regulation system’s model

**Fig. 2:**
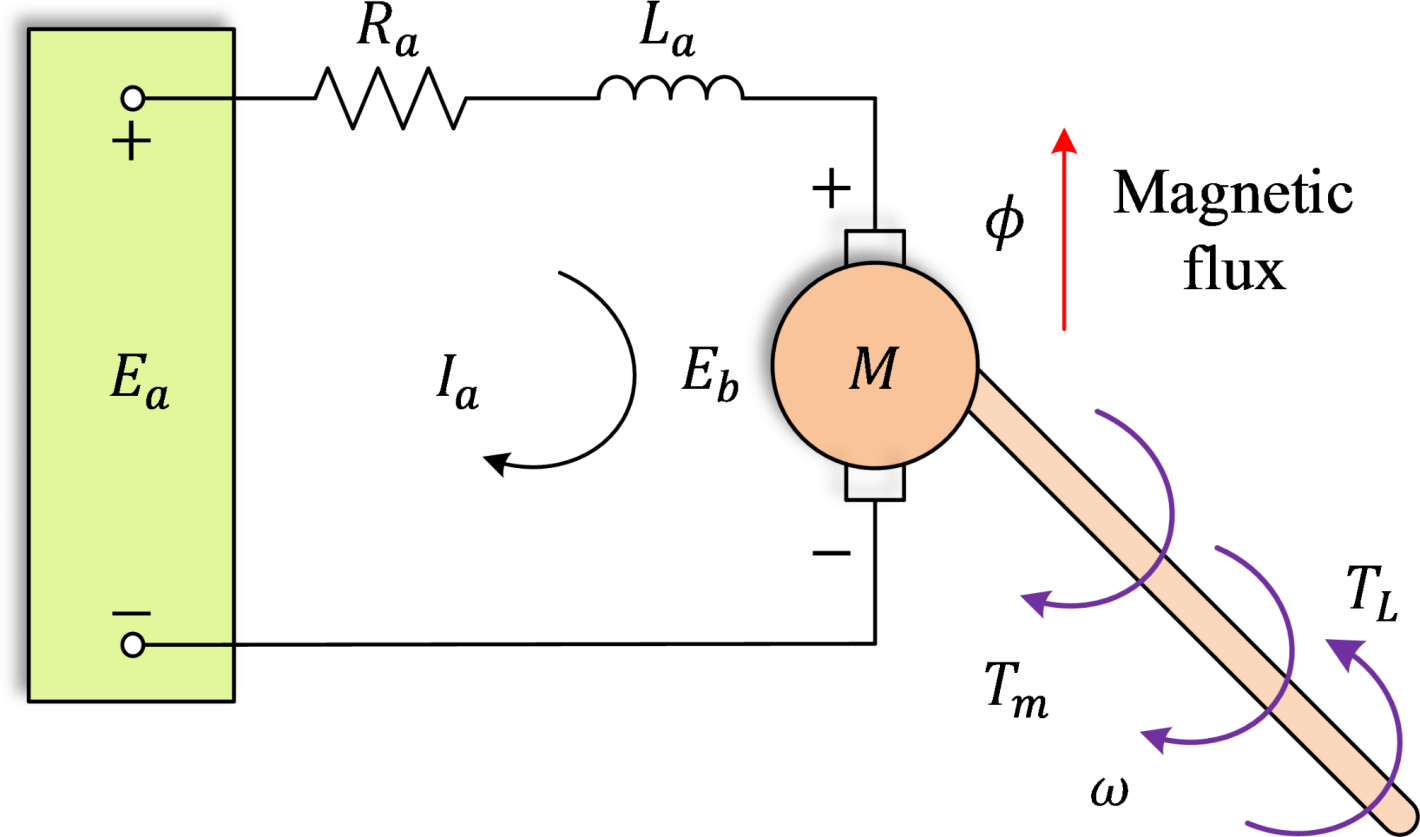
Equivalent DC motor circuit.

The DC motor speed regulation system consists of a DC motor and a mechanical load (as shown in Fig. 2), controlled to regulate speed and torque^48^. A separately excited DC motor is considered in this model. To simplify the system, the following assumptions are made^49^: (1) the DC motor is modeled as a time-invariant linear machine, (2) the mechanical load is represented as a constant torque and (3) the motor is fed with a constant voltage. Based on these assumptions, the system’s dynamics are represented by the following differential equations:

8 $$\:J\left(d\omega\:/dt\right)=\tau\:-B\omega\:$$

9 $$\:{L}_{a}\left(di/dt\right)={E}_{a}-{R}_{a}{I}_{a}-{K}_{e}\omega\:$$

where $\:\tau\:$ is the motor’s torque, $\:\omega\:$ is the angular velocity, $\:{L}_{a}$​ is the motor’s inductance, $\:{R}_{a}$​ is the motor’s resistance, $\:J$ is the moment of inertia, $\:B$ is the damping coefficient, $\:{K}_{e}$​ is the back EMF constant, $\:{E}_{a}$ is the applied voltage, and $\:{I}_{a}$​ is the motor current. The torque $\:\tau\:$ is related to the current by $\:={K}_{t}\cdot\:{I}_{a}$, where $\:{K}_{t}$ is the torque constant. Assuming the motor’s internal dynamics are faster than the mechanical load’s dynamics, the angular velocity $\:\omega\:$ is approximated as constant. Under this assumption, the motor current can be expressed as:

10 $$\:{I}_{a}=\left({E}_{a}-{K}_{e}\omega\:\right)/{R}_{a}$$

This equation indicates that the motor current depends on the applied voltage, resistance, and angular velocity, enabling torque regulation through voltage adjustments. The open-loop transfer function of the DC motor is derived as:

11 $$\:{G}_{motor}\left(s\right)=\frac{{K}_{t}}{\left({L}_{a}s+{R}_{a}\right)\left(Js+B\right)+{K}_{e}{K}_{t}}$$

This model is instrumental for simulating system behavior under varying control strategies and load conditions, supporting the design of control systems tailored to specific applications. For consistency with prior studies, the DC motor parameters are chosen as $\:{R}_{a}=0.4\:{\Omega}$, $\:{L}_{a}=2.7\:\text{H}$, $\:J=0.0004\:kg\cdot\:{m}^{2}$, $\:B=0.0022\:N\cdot\:m\cdot\:s/rad$, $\:{K}_{t}=0.015\:N\cdot\:m/A$, and $\:{K}_{e}=0.05\:V\cdot\:s/rad$^30–33^.

### Three-tanks liquid level system modeling

**Fig. 3:**
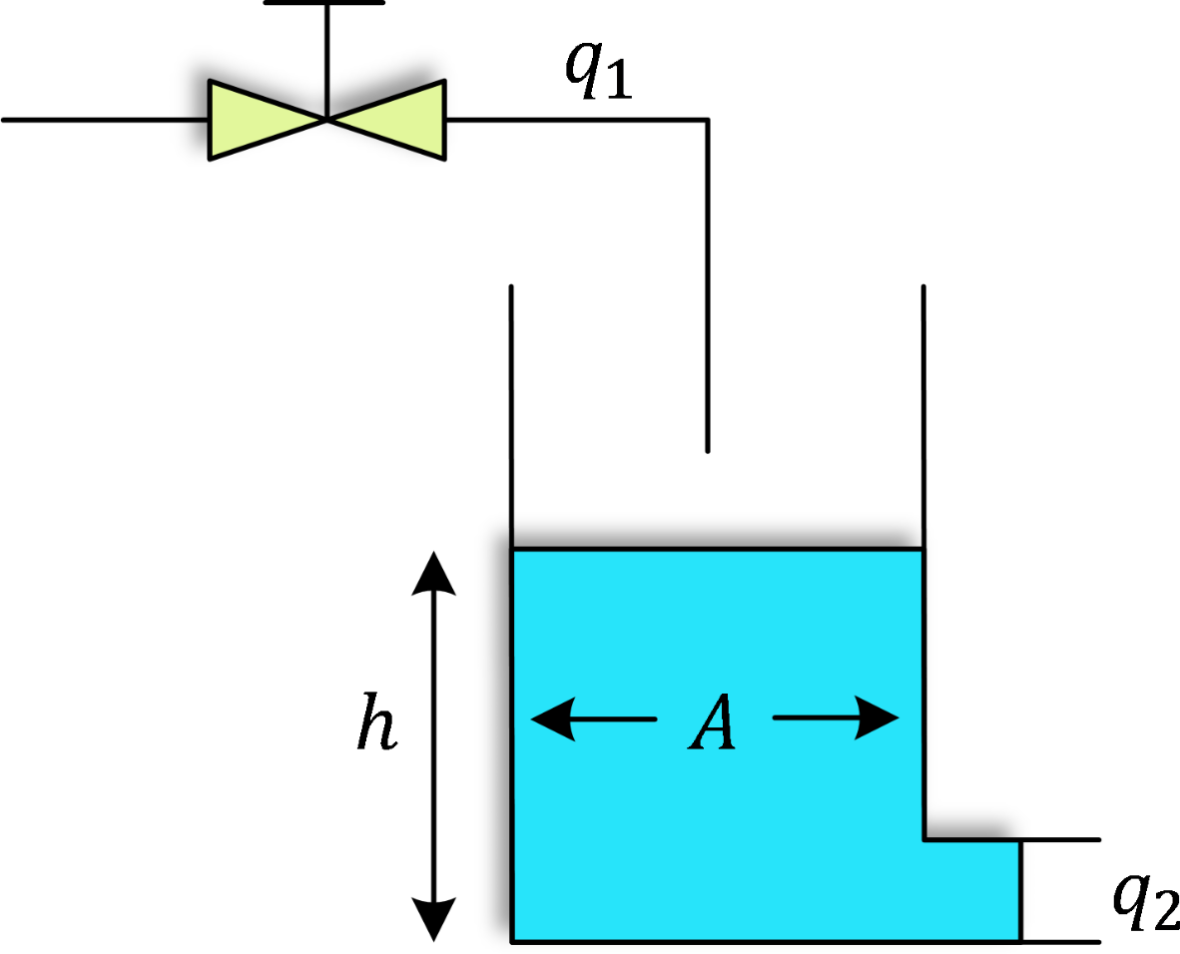
Simple structure of a tank.

The objective of the three-tank liquid level system is to maintain desired liquid levels (as shown in Fig. 3) in Tanks 1, 2, and 3 through a control system. To simplify the model, the following assumptions are made^50,51^: (1) tanks are open to the air, (2) liquid mass is constant and incompressible, (3) flow occurs only from higher to lower levels between tanks, (4) the system is leak-free and (5) flow rates depend on the differences in liquid levels. The dynamics of the system are represented by the following differential equations:

12 $$\:dH1/dt\:=\:qin\:-\:q12\:-\:q13$$

13 $$\:dH2/dt\:=\:q12\:-\:q23$$

14 $$\:dH3/dt\:=\:q13\:+\:q23\:-\:qout$$

Here, $\:H1$, $\:H2$, and $\:H3$​ denote the liquid levels in Tanks 1, 2, and 3, respectively. The flow rates $\:q12$, $\:q13$, and $\:q23$​ are proportional to the differences in liquid levels:

15 $$\:q12\:=\:k12(H1\:-\:H2)$$

16 $$\:q13\:=\:k13(H1\:-\:H3)$$

17 $$\:q23\:=\:k23(H2\:-\:H3)$$

where $\:k12$, $\:k13$, and $\:k23$​ are constants based on the system’s geometry and liquid properties. For analysis, a simplified tank structure is used, where $\:{q}_{1}$ and $\:{q}_{2}$​ are the inflow and outflow rates, $\:h$ represents the liquid height, and $\:A$ is the tank’s cross-sectional area. A transfer function of the form $\:k/\left(Ts+1\right)$​ is applied for consistency with previous studies, where $\:k=0.4$ and $\:T=4$^43,44^. The transfer function for the three-tank liquid level system is expressed as:

18 $$\:{G}_{liquid}\left(s\right)=\frac{1}{64{\text{s}}^{3}+9.6{\text{s}}^{2}+0.48\text{s}+0.008}$$

This model enables simulation of the system’s behavior under various flow rates and control strategies. Control techniques can be applied to adjust flow rates, ensuring the liquid levels meet the requirements of specific applications.

## MGO-based parameter estimation of the controller

**Fig. 4:**
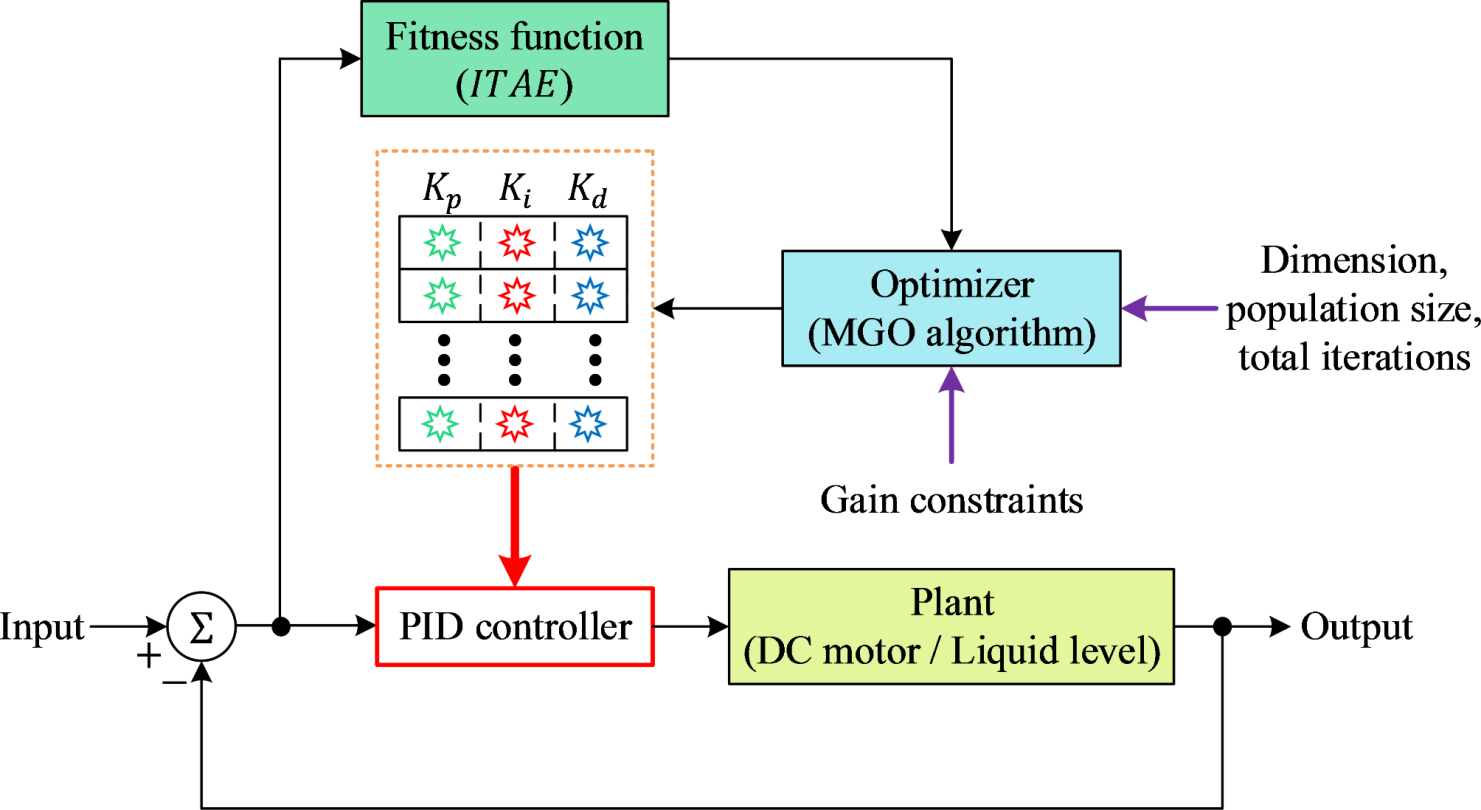
MGO-based PID design framework for DC motor and liquid level systems.

This study utilizes a PID controller to regulate the DC motor and three-tank systems. The PID controller is expressed as:

19 $$\:C\left(s\right)={K}_{p}+\frac{{K}_{i}}{s}+{K}_{d}s$$

where $\:{K}_{p}$, $\:{K}_{i}$, and $\:{K}_{d}$, represent the proportional, integral, and derivative gains, respectively^52^. The control process is illustrated in Fig. 4, where the PID controller operates within a feedback loop to manage the systems. To enable the application of an optimization algorithm, the engineering problems are first formulated as minimization problems.

In this study, the following approach is adopted for describing these systems as minimization problems, where the parameters of the PID controller ($\:\overrightarrow{X}=[{x}_{1},\:{x}_{2},\:{x}_{3}]=[{K}_{p},\:{K}_{i},\:{K}_{d}]$) are optimized using the $\:ITAE$cost function^34^:

20 $$\:ITAE=\underset{0}{\overset{{\infty\:}}{\int\:}}t\cdot\:\left|e\left(t\right)\right|\cdot\:dt$$

Here, $\:e\left(t\right)$ represents the error signal and the minimization problem is subject to the constraint:

21 $$\:0.001\le\:{K}_{p},\:{K}_{i},\:{K}_{d}\le\:20$$

For the optimization task, a population size of 30, a total of 50 iterations, and 25 algorithm runs are considered. Given that the PID controller comprises three parameters, the problem dimension is set to 3. Notably, the MGO algorithm employed is a parameter-free method, eliminating the need for parameter tuning in the proposed optimizer.

## Simulation results and discussion

In this section, we present and discuss the simulation results obtained from applying the MGO-PID controller to the DC motor and three-tank liquid level systems. The results highlight the significant improvements achieved in control performance, such as reduced overshoot, faster response times, and enhanced stability. For the DC motor, the MGO consistently outperforms benchmark algorithms, achieving zero overshoot, rapid convergence, and robust system stabilization. Similarly, for the three-tank system, the MGO demonstrates superior adaptability, minimizing transient fluctuations and ensuring reliable steady-state performance. This discussion not only underscores the algorithm’s practical advantages but also provides insights into its potential for broader applications in dynamic system control.

### Performance of MGO-PID controller on DC motor system

This section evaluates the performance of the MGO-based PID controller for DC motor speed regulation, comparing its efficacy with established algorithms such as the grey wolf optimizer (GWO)^30^, atom search optimization (ASO)^31^, improved model of marine predator algorithm (MMPA)^32^and Henry gas solubility optimization (HGSO)^33^.

**Fig. 5:**
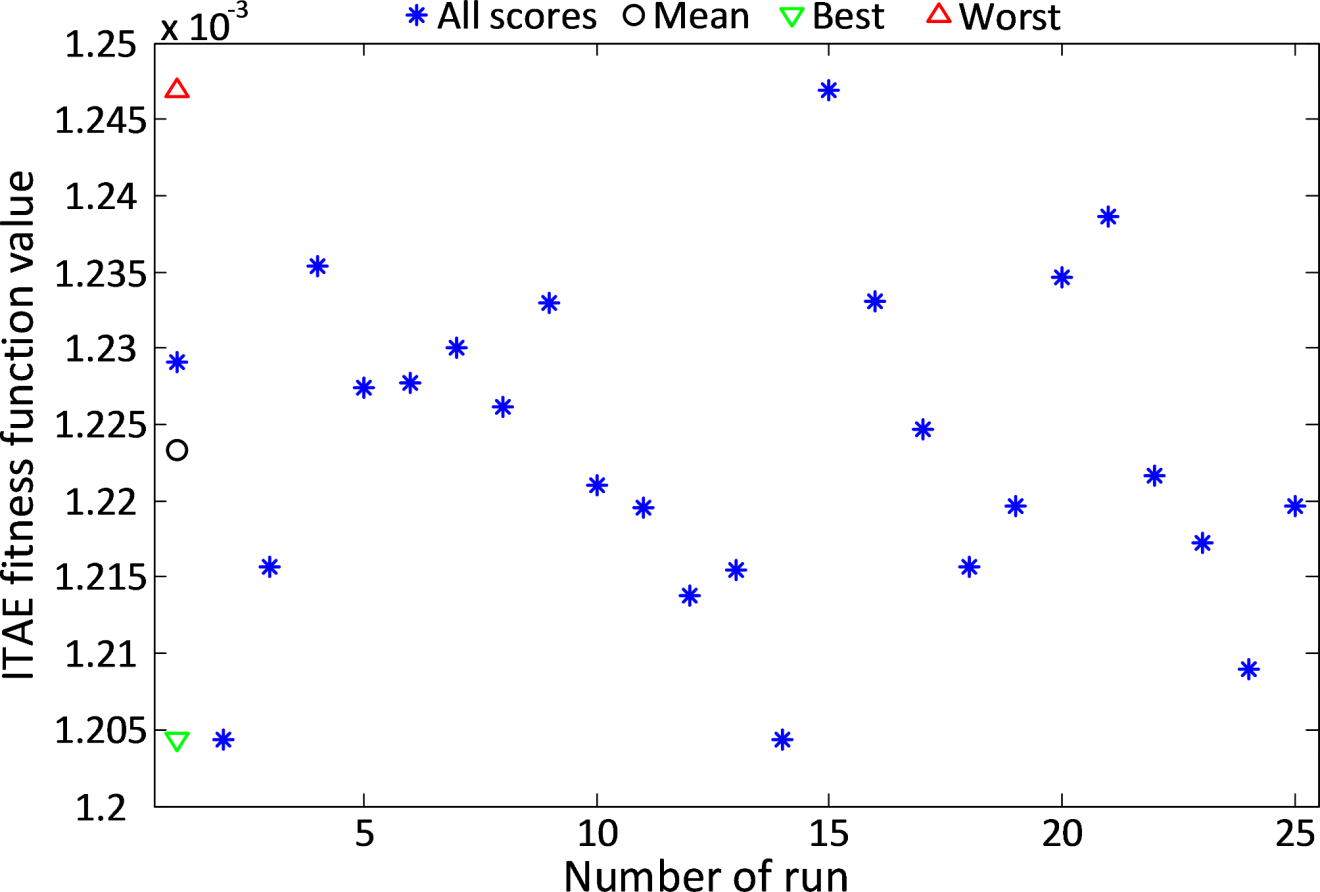
The obtained ITAE values with respect to all runs of MGO-PID controlled DC motor system.

The ITAE values for all runs of the MGO-PID controller are depicted in Fig. 5. These results demonstrate the consistent ability of the MGO algorithm to achieve optimal performance by minimizing the ITAE values across multiple iterations. The narrow range of ITAE values highlights the reliability and robustness of the proposed approach.

**Fig. 6:**
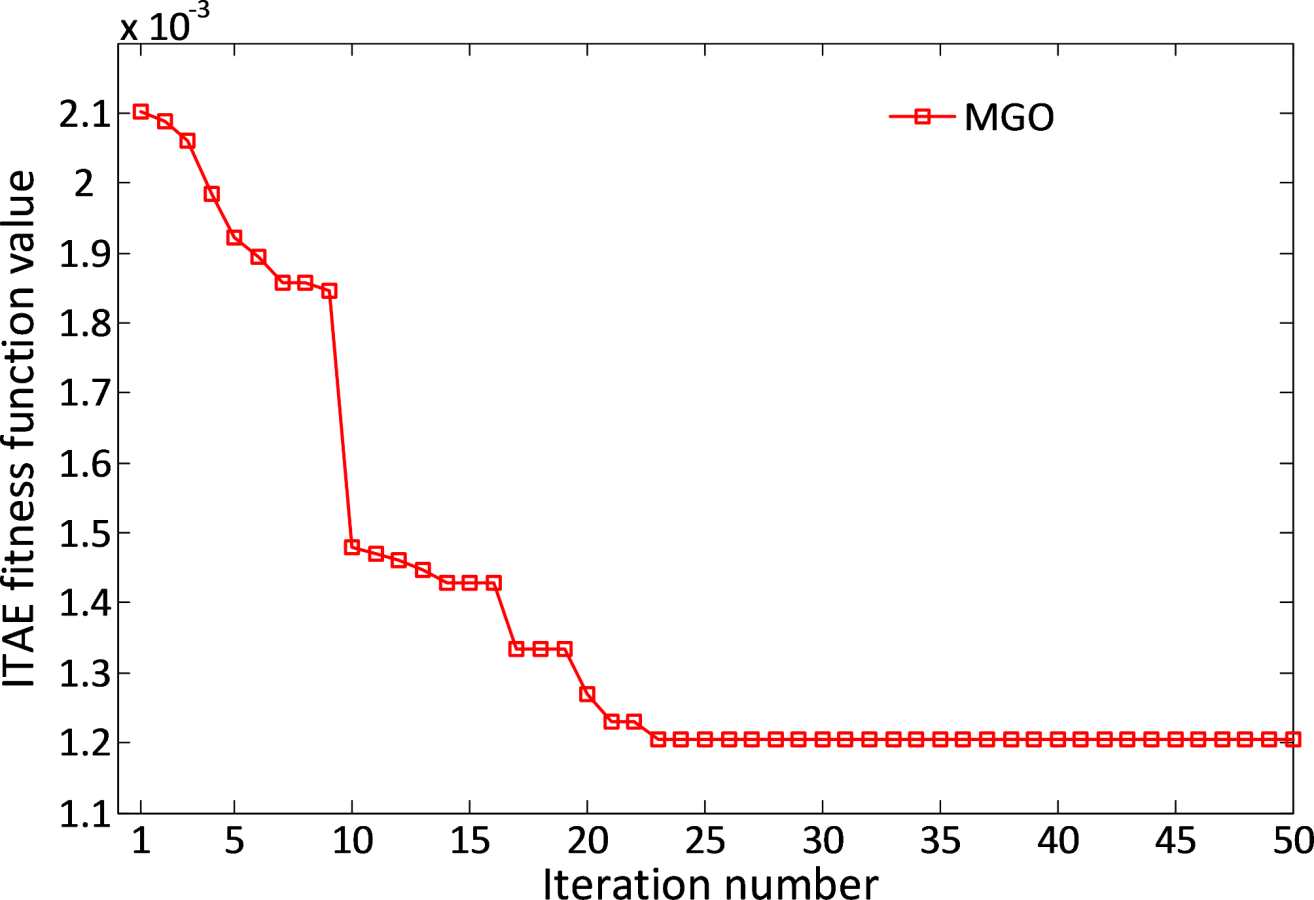
Convergence curve speed of MGO for DC motor system.

Figure 6 illustrates the convergence behavior of the MGO algorithm for the DC motor system. The curve indicates rapid convergence within the initial iterations, signifying the algorithm’s efficiency in reaching an optimal solution. This performance underscores the MGO algorithm’s capability to balance exploration and exploitation effectively during optimization.

The PID parameters obtained via MGO, along with the corresponding performance metrics (overshoot ($\:OS$), rise time ($\:{T}_{r}$), settling time ($\:{T}_{s}$) and peak time ($\:{T}_{p}$)), are summarized in Table 1. For comparison, results from other algorithms (GWO, ASO, MMPA, and HGSO) are also included. The MGO-based controller exhibits the lowest percent overshoot (0%), highlighting superior transient response characteristics compared to GWO (1.5062%), ASO (0%), MMPA (7.0059%), and HGSO (0%). With a rise time of 0.0478 s, the MGO-PID controller achieves the fastest response, outperforming the competing methods. The settling time of the MGO-PID controller (0.0841 s) is significantly lower than that of other algorithms, reflecting its ability to stabilize the system rapidly. The peak time for the MGO approach (0.1506 s) is also the shortest among all algorithms, further affirming its superior control dynamics.

**Table 1:** Obtained PID parameters with different algorithms and corresponding performance metrics for DC motor.

Algorithm	$\:{K}_p$	$\:{K}_{i}$	$\:{K}_{d}$	$\:OS$ (%)	$\:{T}_r$ (sec)	$\:{T}_{s}$ (sec)	$\:{T}_p$ (sec)
MGO (proposed)	18.9937	3.3810	3.2923	**0**	**0.0478**	**0.0841**	**0.1506**
GWO	6.8984	0.5626	0.9293	1.5062	0.1388	0.2052	0.3241
ASO	11.9437	2.0521	2.4358	**0**	0.0692	0.1535	0.9018
MMPA	20	0.7448	1.7395	7.0059	0.0635	0.2793	0.1516
HGSO	13.4430	1.2059	2.2707	**0**	0.0684	0.1186	0.2187

The transfer functions obtained for the DC motor system using the different algorithms are represented in Eqs. (22) to (26). These equations were derived using the optimized PID parameters and confirm the superior tuning capability of the MGO algorithm.

22 $$\:{T}_{MGO}\left(s\right)=\frac{0.04938{s}^{2}+0.2849s+0.05071}{0.00108{s}^{3}+0.05548{s}^{2}+0.2865s+0.05071}$$

23 $$\:{T}_{GWO}\left(s\right)=\frac{0.01394{s}^{2}+0.1035s+0.008439}{0.00108{s}^{3}+0.02004{s}^{2}+0.1051s+0.008439}$$

24 $$\:{T}_{\text{A}\text{S}\text{O}}\left(s\right)=\frac{0.03654{s}^{2}+0.1792s+0.03078}{0.00108{s}^{3}+0.04264{s}^{2}+0.1808s+0.03078}$$

25 $$\:{T}_{\text{M}\text{M}\text{P}\text{A}}\left(s\right)=\frac{0.02609{s}^{2}+0.3s+0.01117}{0.00108{s}^{3}+0.03219{s}^{2}+0.3016s+0.01117}$$

26 $$\:{T}_{\text{H}\text{G}\text{S}\text{O}}\left(s\right)=\frac{0.03406{s}^{2}+0.2016s+0.01809}{0.00108{s}^{3}+0.04016{s}^{2}+0.2033s+0.01809}$$

**Fig. 7:**
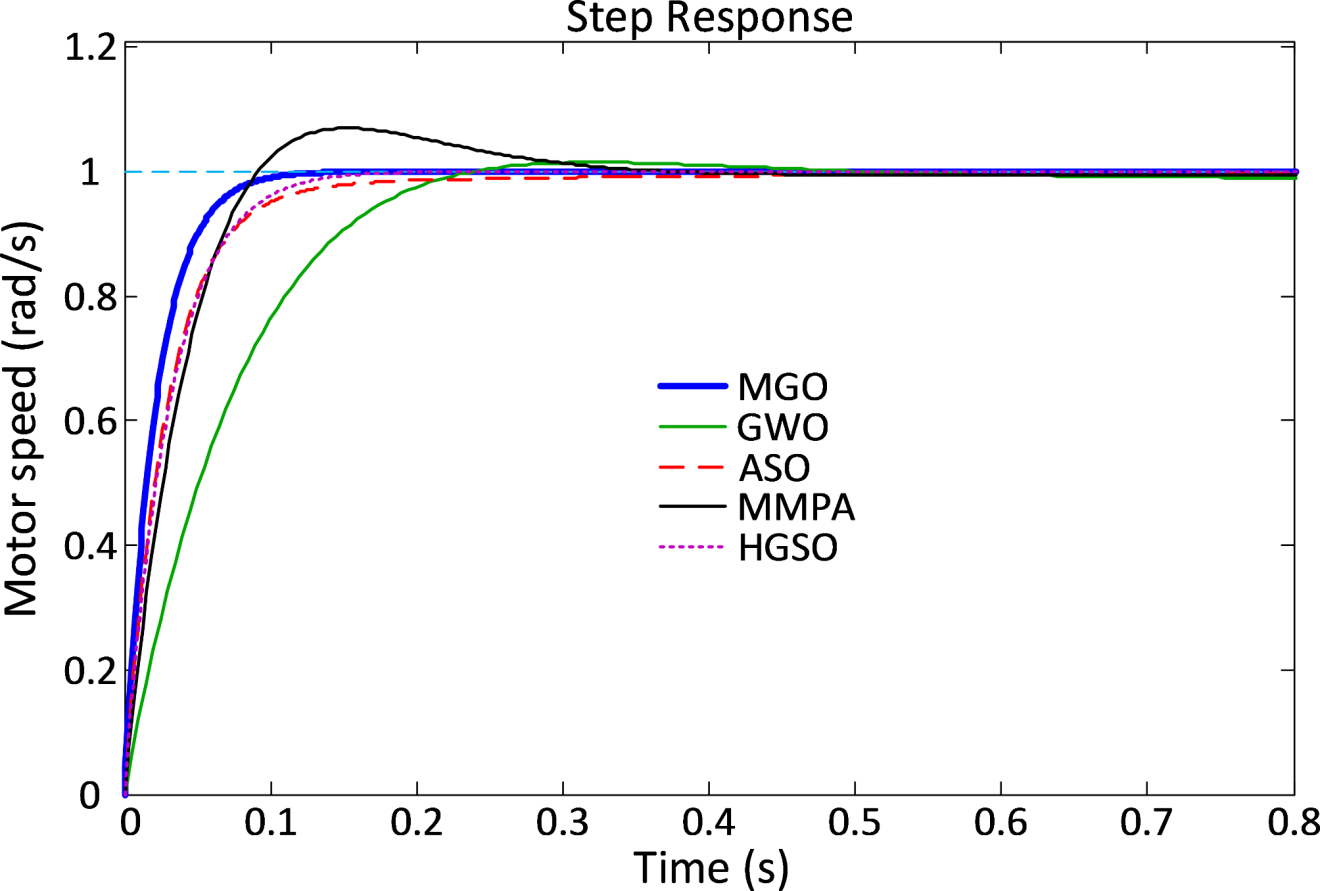
Time response characteristics for DC motor system.

The time-domain response characteristics of the DC motor system under the MGO-PID controller are visualized in Fig. 7. The figure highlights the smooth and rapid transient response of the MGO-based approach, demonstrating its ability to eliminate overshoot and achieve faster stabilization compared to the other methods.

**Fig. 8:**
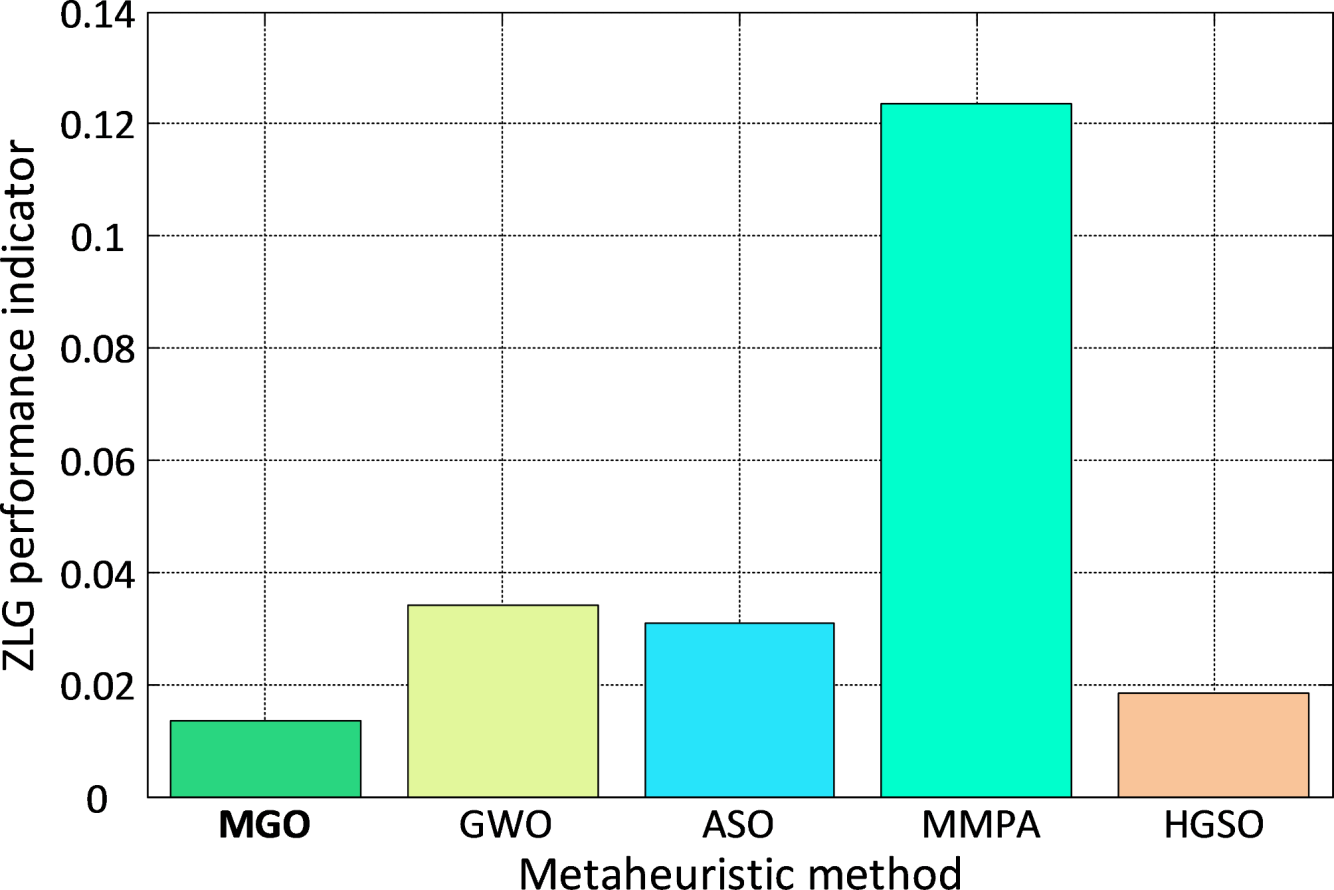
Bar plot of $\:ZLG$ performance indicator for DC motor system.

To further evaluate the control quality, the ZLG^45^ performance indicator, defined in Eq. (27), is employed:

27 $$\:ZLG\left(\overrightarrow{X}\right)=\left(1-{e}^{-\sigma\:}\right)\times\:\left(\frac{OS}{100}+{e}_{ss}\right)+{e}^{-\sigma\:}\times\:({T}_{s}-{T}_{r})$$

where $\:\sigma\:$, $\:OS$, $\:{e}_{ss}$, $\:{T}_{s}$ and $\:{T}_{r}$respectively denote a balancing coefficient, percent overshoot, error (steady state), settling, and rise times^53^. The bar plot in Fig. 8 illustrates the ZLG values for different algorithms. The MGO-based approach achieves the lowest ZLG values, underscoring its superior control performance and adaptability for the DC motor system. In summary, the proposed MGO-PID controller outperforms the benchmark algorithms across multiple performance metrics, offering an enhanced transient response, reduced overshoot, and faster stabilization. These results validate the effectiveness of the MGO algorithm in optimizing control parameters for DC motor speed regulation.

### Performance of MGO-PID controller on liquid level system

This section evaluates the efficacy of the MGO-PID controller in regulating the three-tank liquid level system. The assessment focuses on minimizing the ITAE and compares the results with those obtained using alternative optimization algorithms such as particle swarm optimization (PSO)^43^, hybrid DE and PSO with aging leader and challengers (ALC-PSODE)^43^, covariance matrix adaptation evolution strategy (CMA-ES)^44^and arithmetic optimization algorithm with Harris hawks optimization (AOA-HHO)^44^.

**Fig. 9:**
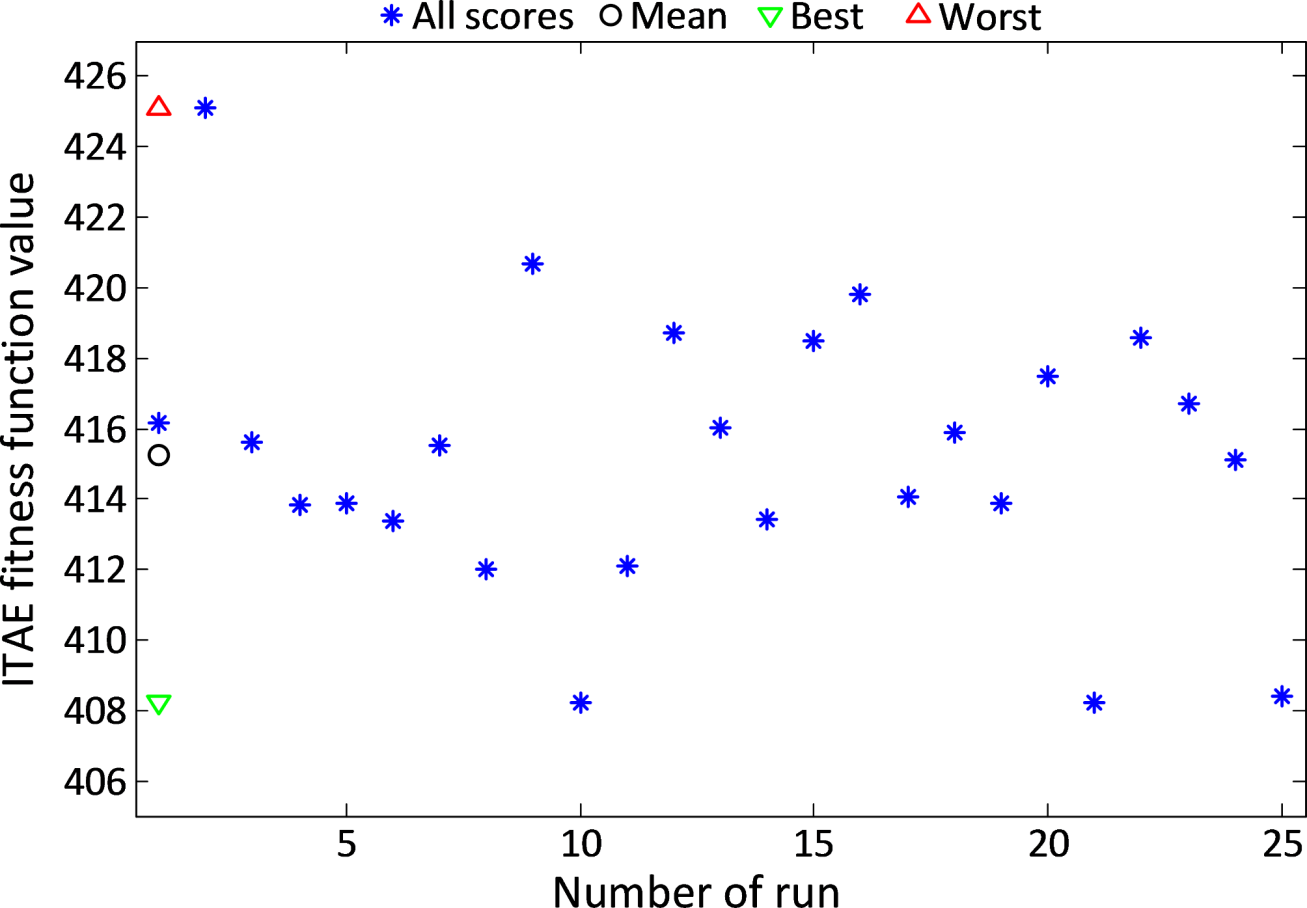
The obtained ITAE values with respect to all runs of MGO-PID controlled liquid level system.

Figure 9 displays the ITAE values achieved during all runs of the MGO-PID controller for the liquid level system. These results indicate a consistently narrow range of ITAE values, showcasing the robustness and reliability of the MGO algorithm in optimizing control performance.

**Fig. 10:**
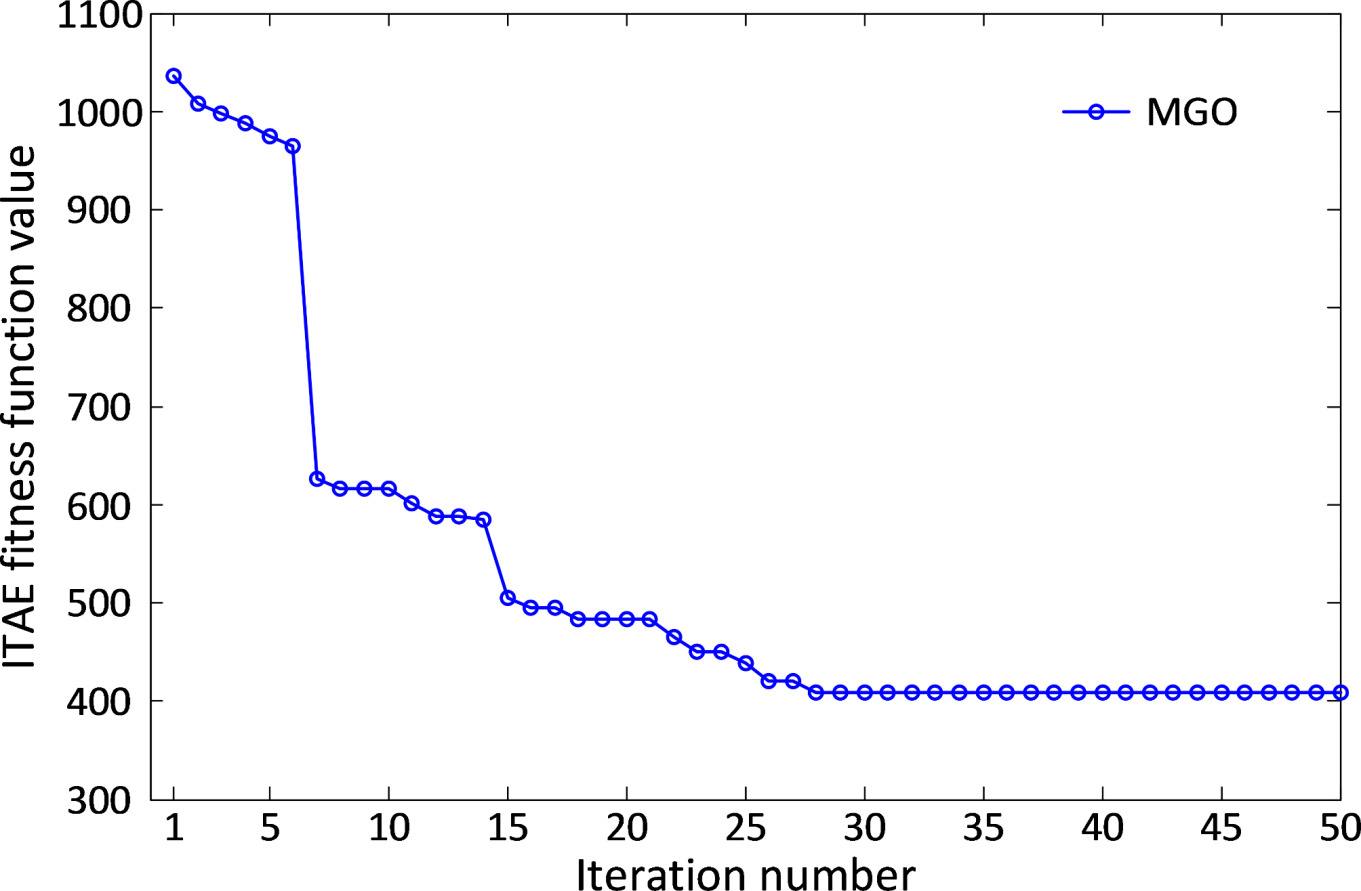
Convergence curve speed of MGO for liquid level system.

The convergence characteristics of the MGO algorithm for the liquid level system are illustrated in Fig. 10. The algorithm demonstrates swift convergence within the first 29 iterations, emphasizing its capability to achieve optimal performance in minimal time. This highlights the MGO algorithm’s efficiency in balancing exploration and exploitation during the optimization process.

The optimal PID parameters and corresponding performance metrics ($\:OS$, $\:{T}_{r}$, $\:{T}_{s}$ and $\:{T}_{p}$) for the liquid level system are summarized in Table 2. The results obtained via the MGO-PID controller are compared to those of competing algorithms. The MGO-PID controller achieves an overshoot of 12.0927%, which is notably lower than that of CMA-ES (49.9912%) and AOA-HHO (20.1160%), highlighting its superior transient performance. With a rise time of 11.0424 s, the MGO-PID controller demonstrates a competitive response time, outperforming CMA-ES (15.0133 s) and AOA-HHO (17.7926 s). The settling time of 60.6037 s for the MGO-PID controller is substantially better than those of CMA-ES (238.6552 s) and AOA-HHO (160.1051 s), reflecting enhanced system stability. The MGO-based approach achieves a peak time of 22.9162 s, marking a considerable improvement over competing methods.

**Table 2:** Obtained PID parameters with different algorithms and corresponding performance metrics for liquid level.

Algorithm	$\:{K}_p$	$\:{K}_{i}$	$\:{K}_{d}$	$\:OS$ (%)	$\:{T}_r$ (sec)	$\:{T}_{s}$ (sec)	$\:{T}_p$ (sec)
MGO (proposed)	0.04168	0.00094	1.31334	**12.0927**	**11.0424**	**60.6037**	**22.9162**
PSO	0.0528	0.0003	1	14.4337	12.2179	320.5526	26.3215
ALC-PSODE	0.0419	0.0009	1	12.4585	12.8263	64.2188	26.3976
CMA-ES	0.051	0.0013	0.3914	49.9912	15.0133	238.6552	38.2793
AOA-HHO	0.040	0.0005	0.4269	20.1160	17.7926	160.1051	39.2792

The transfer functions derived for the liquid level system using the MGO algorithm and alternative approaches are represented in Eqs. (28) to (32). These equations validate the superior tuning capabilities of the MGO algorithm in optimizing the system’s parameters.

28 $$\:{T}_{MGO}\left(s\right)=\frac{1.313{s}^{2}+0.04168s+0.00094}{64{s}^{4}+9.6{s}^{3}+1.793{s}^{2}+0.04968s+0.00094}$$

29 $$\:{T}_{PSO}\left(s\right)=\frac{{s}^{2}+0.0528s+0.0003}{64{s}^{4}+9.6{s}^{3}+1.48{s}^{2}+0.0608s+0.0003}$$

30 $$\:{T}_{\text{A}\text{L}\text{C}-\text{P}\text{S}\text{O}\text{D}\text{E}}\left(s\right)=\frac{{s}^{2}+0.0419s+0.0009}{64{s}^{4}+9.6{s}^{3}+1.48{s}^{2}+0.0499s+0.0009}$$

31 $$\:{T}_{\text{C}\text{M}\text{A}-\text{E}\text{S}}\left(s\right)=\frac{0.3914{s}^{2}+0.051s+0.0013}{64{s}^{4}+9.6{s}^{3}+0.8714{s}^{2}+0.059s+0.0013}$$

32 $$\:{T}_{\text{A}\text{O}\text{A}-\text{H}\text{H}\text{O}}\left(s\right)=\frac{0.4269{s}^{2}+0.04s+0.0005}{64{s}^{4}+9.6{s}^{3}+0.9069{s}^{2}+0.048s+0.0005}$$

**Fig. 11:**
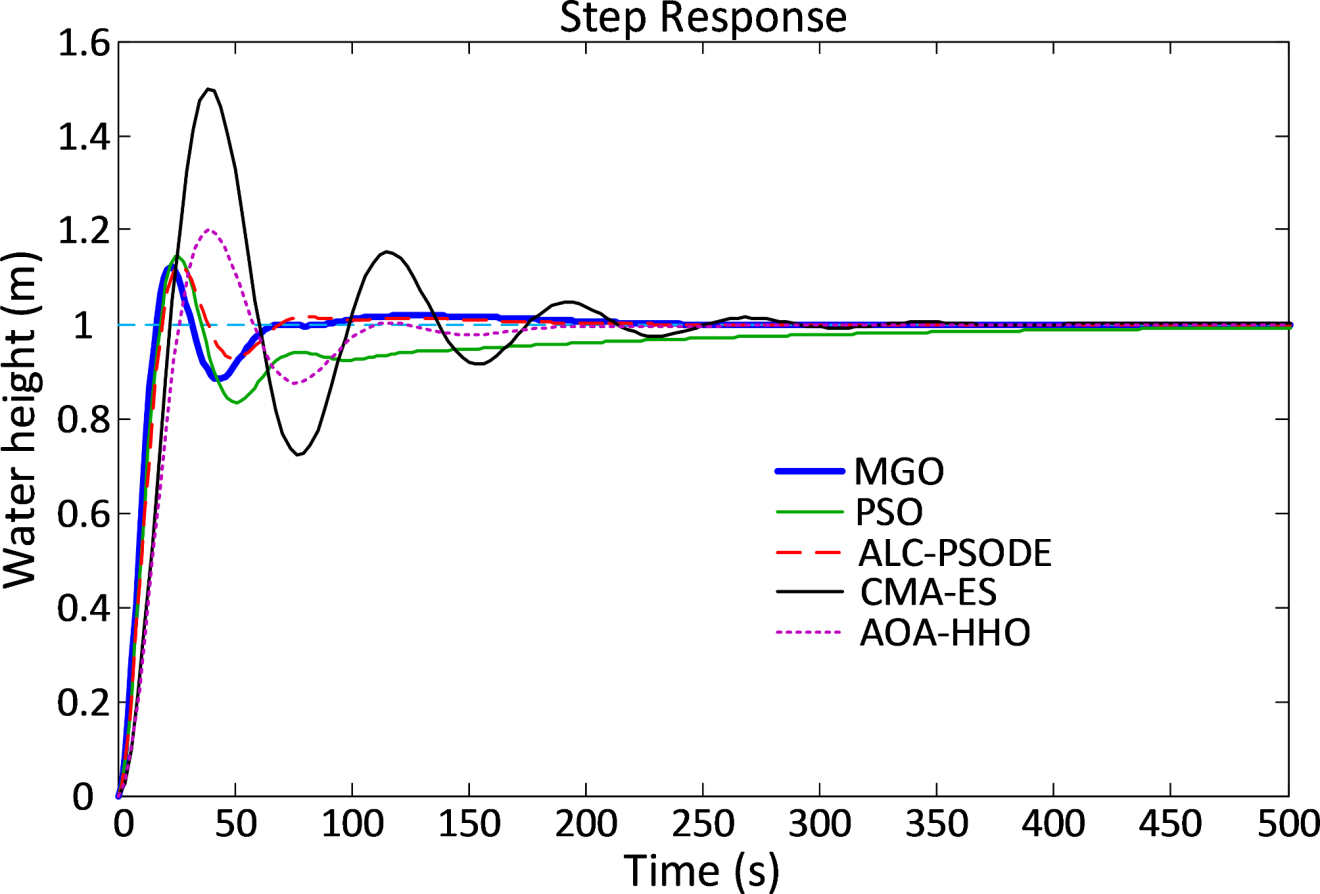
Time response characteristics of liquid level system.

Figure 11 illustrates the time response characteristics of the liquid level system for various algorithms. The MGO-PID controller exhibits a smoother and more stable response, with minimized overshoot and faster stabilization, further reinforcing its efficacy.

**Fig. 12:**
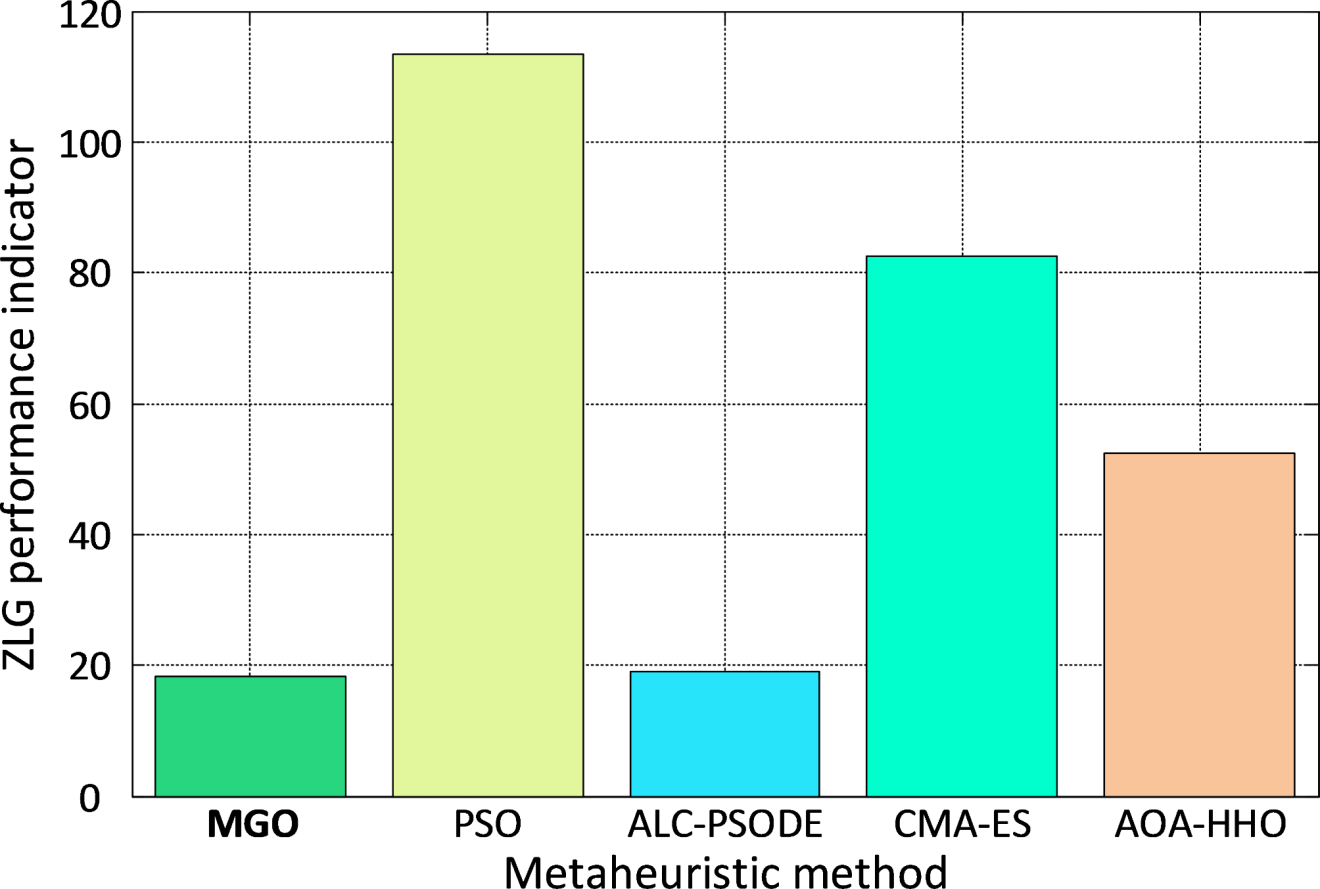
Bar plot of $\:ZLG$ performance indicator for liquid level system.

To comprehensively evaluate the controller’s performance, the ZLG indicator, as defined in Eq. (27), is employed. Figure 12 presents a comparative bar plot of ZLG values for different algorithms. The MGO-PID controller achieves the lowest ZLG value, emphasizing its effectiveness in ensuring optimal control quality for the liquid level system. In summary, the MGO-PID controller demonstrates significant improvements over existing algorithms, achieving lower overshoot, faster convergence, and superior stability in the liquid level system. These results underscore the robustness and adaptability of the MGO algorithm for optimizing control parameters in complex dynamic systems.

## Conclusion

This study introduces a novel and efficient method for optimizing the control parameters of DC motor and three-tank liquid level systems by employing the MGO. Inspired by the swift and strategic behaviors of mountain gazelles, the MGO is leveraged to enhance control performance in these systems. The proposed approach is thoroughly evaluated through comparisons with several state-of-the-art algorithms, including the grey wolf optimizer, atom search optimization, improved model of marine predator algorithm, Henry gas solubility optimization, particle swarm optimization, hybrid differential evolution and particle swarm optimization with aging leader and challengers, covariance matrix adaptation evolution strategy, and arithmetic optimization algorithm with Harris hawks optimization. The results highlight the MGO’s superior ability to optimize control parameters, as evidenced by its consistently better time-domain performance metrics. These include reduced overshoot, faster rise times, shorter settling times, and minimized peak times. Such improvements directly enhance the stability, efficiency, and precision of the controlled systems, demonstrating the practical advantages of adopting the MGO-based approach for real-world applications. In addition, this paper introduces the ZLG performance indicator, a novel metric for evaluating control quality comprehensively. The MGO-based method achieves significantly lower ZLG values compared to the competing algorithms, further validating its effectiveness. This underscores the robustness and adaptability of the MGO in addressing optimization challenges and maintaining superior control over dynamic systems. Through this detailed analysis, the findings emphasize the potential of the MGO algorithm as a powerful tool for optimizing control parameters, offering a reliable and efficient solution for complex engineering problems such as those found in DC motor and liquid level control systems.

While the MGO demonstrates significant advantages in terms of fast convergence, robustness, and effective balancing of exploration and exploitation, certain limitations remain. First, the algorithm’s performance may degrade when applied to highly complex, high-dimensional problems due to its reliance on predefined parameters. Although MGO has shown superior performance compared to other algorithms in this study, further benchmarking against more diverse datasets and larger-scale systems is necessary to establish its generalizability. Additionally, as with many metaheuristic algorithms, there is no theoretical guarantee of finding the global optimum, and the performance can be influenced by the problem-specific landscape. Lastly, computational efficiency for real-time applications could be a concern, particularly for systems requiring rapid iterations or low latency. These limitations highlight the scope for future research, such as improving the scalability of MGO, incorporating adaptive parameter mechanisms, or hybridizing MGO with other techniques to enhance its robustness and efficiency across diverse applications.

## Data Availability

Data is available from the authors upon reasonable request from Absalom E. Ezugwu.
